# Galectin-3 Release in the Bone Marrow Microenvironment Promotes Drug Resistance and Relapse in Acute Myeloid Leukemia

**DOI:** 10.3390/life15060937

**Published:** 2025-06-10

**Authors:** Cansu Yıldırım

**Affiliations:** Atatürk Vocational School of Health Services, Afyonkarahisar Health Sciences University, 03030 Afyonkarahisar, Türkiye; cansu.yalcin@afsu.edu.tr

**Keywords:** Galectin-3, bone marrow, AML, MSC, survival, drug resistance, relapse

## Abstract

Reciprocal signaling between acute myeloid leukemia (AML) cells and the surrounding bone-marrow microenvironment (BMME) promotes AML progression through several mechanisms. One of the most important mechanisms is the induction of Galectin-3 (Gal-3) expression by AML cells and bone marrow mesenchymal stromal cells (BM-MSCs). Emerging evidence indicates that Gal-3 upregulation in the BMME promotes AML cell adhesion and survival, leading to the development of chemotherapy resistance, relapse, and poor prognosis. Identifying the biological function and critical signaling pathways of Gal-3 may contribute to overcoming acquired drug resistance and preventing post-treatment relapse. Gal-3 is involved in several molecular signaling pathways, including PI3K/AKT/mTOR, Ras/Raf/MEK/ERK, JAK/STAT, JNK, Wnt/β-catenin, PLC/PKC and NF-κB, which are interconnected to promote AML cell survival and resistance to chemotherapy. This review focuses on the biological effects, molecular mechanisms of action and regulation of Gal-3 in the pathogenesis and progression of AML. The therapeutic potential of potent synthetic small-molecule Gal-3 inhibitors in high-risk patients with AML is also discussed based on preclinical and clinical evidence from several human diseases. Currently, the effect of these Gal-3 inhibitors in AML has not been investigated either in vitro or in vivo. The findings provide a rationale for targeting Gal-3 that may be a very promising therapeutic approach, especially for patients with relapsed/refractory AML, and may enhance the efficacy of conventional chemotherapeutic drugs and/or immune checkpoint inhibitors.

## 1. Introduction

The bone-marrow microenvironment (BMME) serves as a haven for leukemic cells. BMME mediates drug resistance of leukemic cells and ultimately leads to disease relapse. Leukemic cells are supported by stromal cells, such as mesenchymal stromal cells (MSCs), in the bone marrow (BM) [1,2,3,4], but also by the extracellular matrix (ECM) [5,6]. It has been observed that changes in BM-MSCs may contribute to the progression of acute myeloid leukemia (AML) by expressing and/or releasing certain factors [7]. By understanding the mechanism by which the BM niche helps AML cells and developing approaches that inhibit this underlying mechanism, we can improve patient outcomes. The pathophysiology of AML is complex and existing treatments are inadequate. Therefore, to create a successful treatment, a deeper understanding of the underlying process of AML is required.

Previous studies have shown that galectin-3 (Gal-3) expression plays a role in many hematological malignancies [8,9,10,11,12]. Gal-3 is a chimera-type member of the β-galactoside-binding lectin family of proteins encoded by the LGALS3 gene located at locus q21-q22 of chromosome 14 in humans. This protein has a 12 amino acid N-terminal region (NTR) with a serine (S) 6 phosphorylation site that mediates its translocation from the nucleus to the cytoplasm, followed by a 100 amino acid collagen-like sequence (CLS) domain that can be cleaved by matrix metalloproteases (MMPs) and a single 130 amino acid long carbohydrate-recognition domain (CRD) at the C-terminus containing the NWGR anti-death motif of the bcl-2 family. The N-terminal proline- and glycine-rich domain of Gal-3 can form a multimer, which enables this Gal to crosslink and form lattice-like structures between glycan-containing molecules on diverse cell surfaces and within the ECM [13,14,15,16,17,18,19]. Gal-3 is widely distributed in human non-hematopoietic and hematopoietic tissues [20], including immune cells, such as monocytes and macrophages [21], dendritic cells [22], neutrophils [23], eosinophils [24], basophils [25], mast cells [25] and NK cells [26], with the exception of T and B cells [27,28]. Gal-3 is expressed in various cellular compartments, including the nucleus, cytoplasm, mitochondria, and cell surface. However, it is also found in the extracellular space and circulation and is secreted via non-classical secretory pathways due to the lack of a secretory signal sequence [15,19,29,30,31,32,33,34]. Gal-3 can be secreted via exosomes, vesicular release, or by crossing the lipid bilayer [19,30]. Gal-3 performs both intracellular and extracellular functions by interacting with multiple ligands, thereby affecting various signaling pathways. It is known that the subcellular localization of Gal-3 determines its function. The location and function of Gal-3 are regulated by post-translational modifications, such as serine and threonine (T) phosphorylation [35,36,37,38]. As a multifunctional protein, Gal-3 is crucial for cell survival and cell cycle progression in the cytoplasm, while in the nucleus it stimulates pre-mRNA splicing and regulates gene transcription. In the mitochondria, it protects mitochondrial integrity by inhibiting cytochrome c release, and in the extracellular environment, it modulates cell–cell and cell–matrix adhesions, playing a crucial role in cancer progression and metastasis [19,33,39,40,41,42,43]. Interestingly, Gal-3 is highly expressed and secreted in patients with AML, and associated with poor prognosis [8,44,45,46].

Therefore, this review describes the current findings regarding the function, molecular mechanisms of action and regulation of Gal-3 in the pathogenesis and progression of AML, with special emphasis on its role in BMME-induced chemoresistance. Finally, the therapeutic potential of potent synthetic small-molecule Gal-3 inhibitors in human diseases is discussed, providing a reference for the application of the relevant Gal-3 inhibitor in the treatment of patients with AML, especially in patients with relapsed or refractory AML.

## 2. Acute Myeloid Leukemia

### 2.1. High BM Gal-3 Expression and Plasma Gal-3 Levels Are Associated with Poor Prognosis

**Figure 1:**
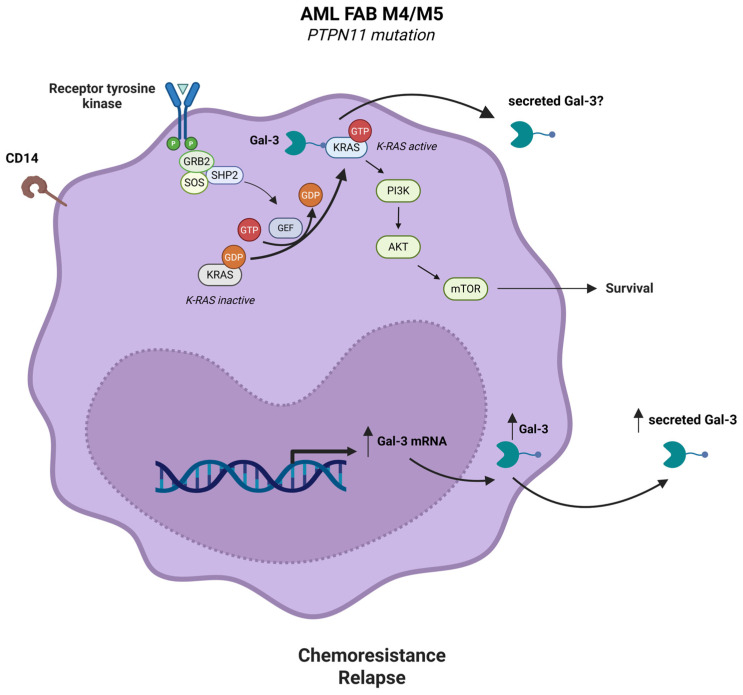
High Gal-3 mRNA and protein expression in AML cells and its secretion in the BMME leads to AML cell survival, chemoresistance and relapse. High Gal-3 expression is strongly related to AML FAB M4/M5 subtypes, CD14 expression and PTPN11 mutation. Somatic gain-of-function mutations of PTPN11 are found in patients with AML FAB M4/M5 subtypes. PTPN11 gene encodes the cytoplasmic protein tyrosine phosphatase SHP-2, which stimulates RAS signaling. Gal-3 may interact with active K-Ras in the plasma membrane and may stabilize K-Ras in its active state, which may activate the pro-survival PI3K/AKT/mTOR signaling. Created with Biorender.com.

Several studies have investigated the prognostic significance of Gal-3 expression in AML. Cheng et al. examined LGALS3 mRNA expression in mononuclear cells from bone marrow (BM) of patients with newly diagnosed non-acute promyelocytic leukemia (non-APL; non-M3 AML) before treatment and correlated the findings with the patients’ clinical characteristics and outcomes. The median value of BM LGALS3 mRNA expression in these patients was used as a cut-off point to distinguish between groups with lower and higher expression. Higher LGALS3 mRNA expression was strongly related to older age, AML French-American-British (FAB) M4/M5 subtypes, leukemic CD14 expression and PTPN11 mutation, while it was negatively associated with FAB AML M1 subtype, blast counts, serum lactate dehydrogenase (LDH), FLT3-ITD and CEBPA mutations [8]. The result that higher LGALS3 mRNA expression was strongly connected with PTPN11 mutation was intriguing. The PTPN11 gene encodes the cytoplasmic protein tyrosine phosphatase SHP-2, which positively modulates rat sarcoma (RAS) signaling [47]. Somatic gain-of-function mutations of PTPN11 are identified in patients with myelodysplastic syndrome (MDS), juvenile myelomonocytic leukemia, and de novo AML, especially in the FAB M4/M5 subtypes [48,49]. It has been demonstrated that Gal-3-mediated cell transformation is partly due to its interaction with active K-Ras in the cell membrane, the most important Ras oncoprotein in human tumors [50]. Gal-3 may bind membrane-bound active K-Ras protein in patients with PTPN11 mutations, which may lead to translocation of cytosolic Gal-3 to the plasma membrane. Gal-3 binding to active K-Ras may render K-Ras less sensitive to Ras-GTPase-activating proteins (GAPs), thereby stabilizing K-Ras in its active state, which is necessary for K-Ras signaling and thereby activates phosphatidylinositol-3 kinase (PI3K) [50] (Figure 1).

BM Gal-3 protein expression and plasma levels correlated significantly with BM LGALS3 mRNA expression in patients with AML. BM Gal-3 protein was present in the cytosol and nucleus of leukemic cells. Patients with AML with higher LGALS3 mRNA expression had higher primary refractory rates to chemotherapy, lower complete remission (CR) rates and shorter overall survival (OS) compared to those with lower LGALS3 mRNA expression. Higher BM LGALS3 mRNA expression was an independent unfavorable prognostic factor for OS in non-M3 AML and cytogenetically normal (CN) patients with AML. Interestingly, in patients undergoing allogeneic hematopoietic stem cell transplantation (HSCT), the adverse prognostic effect of higher BM LGALS3 mRNA expression on OS was lost, which suggests that HSCT may reduce the adverse effects of higher BM LGALS3 mRNA expression on survival. Despite the small patient population in the independent validation cohort, the unfavorable prognostic impact of higher BM LGALS3 mRNA expression was confirmed in that cohort. The underlying mechanisms connecting higher LGALS3 mRNA expression with unfavorable AML prognosis remain to be uncovered [8].

Gao et al. investigated the clinical significance of plasma Gal-3 levels in AML. Peripheral blood (PB) samples of a group of patients with de novo non-M3 AML and normal donors were recruited to measure the plasma Gal-3 levels. The plasma Gal-3 median level was significantly higher in non-M3 patients with AML compared to normal donors. A comparison between several non-M3 AML FAB subtypes showed that the plasma Gal-3 median levels were significantly higher in the M4/M5 subtypes than in the M1/M2 subtypes. They also found that the plasma Gal-3 median levels of de novo and relapsed non-M3 patients with AML were primarily higher than those of the non-M3 patients with AML during CR, which suggests that Gal-3 might be essential in the minimal residual disease (MRD) maintenance. Next, the plasma Gal-3 median value in the total number of non-M3 patients with AML was taken as a cut-off point to divide the patients into two groups: lower- and higher plasma Gal-3 level groups. In patients with higher plasma Gal-3 levels, serum LDH levels were lower, and they were older than the patients with lower plasma Gal-3 levels. Furthermore, patients with higher plasma Gal-3 levels had a higher frequency of cytogenetic aberrations. Regarding the molecular abnormalities, a negative correlation between CEBPA^double-mutation^ and higher plasma Gal-3 levels was observed [45]. CEBPA is a transcription factor that regulates the differentiation of myeloid progenitors. Mutated CEBPA is an important prognostic marker in AML. CEBPA^double-mutation^ has been associated with a favorable prognosis in patients with AML. CEBPA^double-mutation^ refers to a double mutation that occurs in two different alleles of the CEBPA gene in AML. These biallelic mutations frequently consist of an N-terminal mutation on one allele and a C-terminal basic leucine zipper (bZIP) gene mutation on the other. These mutations typically involve premature stop codons, often caused by frameshift alterations in the protein’s N-terminal region, as well as in-frame mutations at the C-terminal bZIP domain that disrupt DNA binding and/or the formation of homo- and heterodimers. The combination of N- and C-terminal/bZIP mutations disrupts normal CEBPA function and downstream cellular processes, such as cell cycle arrest and myeloid differentiation [51,52,53]. Compared to patients with lower plasma Gal-3 levels, those with higher plasma Gal-3 levels exhibited lower rates of CR and 1-year OS. The Kaplan–Meier survival analysis revealed that the OS was considerably shorter in the group with higher plasma Gal-3 levels. A multivariate cox proportional hazards model showed that a higher plasma Gal-3 level was an independent unfavorable prognostic factor for OS in non-M3 AML and CN patients with AML [45]. Overall, higher BM Gal-3 expression and plasma Gal-3 are independent unfavorable prognostic biomarkers for OS in non-M3 AML and CN patients with AML. Therefore, Gal-3 may serve as a potential therapeutic target in patients with AML with higher expression levels of this protein.

Acute promyelocytic leukemia (APL) is an AML subtype that is characterized by a balanced reciprocal translocation t(15;17)(q22;21), resulting in the development of the promyelocytic leukemia-retinoic acid receptor α (PML-RARα) fusion gene. APL patients who receive all-trans retinoic acid (ATRA) and arsenic trioxide treatment have a good prognosis [54]. Nonetheless, relapse and early mortality continue to be the largest threats to long-term survival in APL patients [55,56]. Gao et al. also examined the prognostic significance of serum Gal-3 levels in newly diagnosed APL patients. APL patients had higher median Gal-3 levels compared to the healthy controls. Next, they separated the APL patients into two subgroups: those with higher and lower serum Gal-3 levels, using the median value of the Gal-3 level as a cut-off point. Higher median Gal-3 levels were closely related to older age, coagulopathy, psoriasis and CD34 expression. The Kaplan–Meier analysis showed that APL patients with higher Gal-3 levels had significant shorter OS and relapse-free survival (RFS), and higher cumulative incidence of relapse than those with lower Gal-3 levels. This demonstrated that higher Gal-3 levels may be linked with early recurrence in APL patients [44]. Therefore, it is possible that higher Gal-3 levels may upregulate CD34 expression in relapsing APL patients. There may be a shift towards a more immature phenotype and a possible expansion of the leukemia-initiating compartment in patients with relapsed APL, as evidenced by the fact that they had higher CD34 expression compared to de novo APL patients [57]. Furthermore, the Kaplan–Meier analysis for OS also showed that APL patients with the highest Gal-3 levels had the worst prognosis. A multivariate analysis by Cox proportional hazards model showed that high Gal-3 levels were the only independent adverse risk factor for RFS in APL patients treated with ATRA and arsenic trioxide-based frontline therapy. In conclusion, high serum Gal-3 levels are also an independent adverse prognostic factor in APL patients and may become a reliable biomarker to predict relapse in APL patients [44].

One of the most prevalent chromosomal abnormalities in adult AML is t(8;21)(q22;q22). It is thought that AML with t(8;21)/AML1-ETO has a good prognosis. Nevertheless, the clinical outcome of these patients with AML varies, with recurrence reported in about 30% of cases [58,59]. To ensure that t(8;21) patients with AML can receive personalized treatment, Wang et al. searched for possible predictive risk factors. They showed that higher serum Gal-3 levels were related to a higher cumulative incidence of recurrence (CIR) in patients with AML with t(8;21), suggesting that a high serum Gal-3 level is associated with relapse in these patients. Moreover, according to univariate analysis, high serum Gal-3 negatively impacted OS and disease-free survival (DFS) in patients receiving standard-dose cytarabine-based consolidation chemotherapy. Multivariate analysis revealed that Gal-3 was an independent factor that negatively impacted OS. Interestingly, BM CD56 expression was significantly higher in patients with higher serum Gal-3 levels than in those with lower serum Gal-3 levels [46]. CD56 expression has been identified as a positive predictive factor for relapse in t(8;21) patients with AML [60]. Wang et al. also showed that t(8;21) patients with AML with CD56 expression had significantly higher relapse rate and CIR, and had significant shorter OS and DFS [46]. Constitutive activation of the MAPK pathway, which contributes to Gal-3 gene overexpression [61], is intimately linked to CD56 expression [62]. Through the activation of MAPK signaling, increased Gal-3 expression promotes tumor cell growth and invasiveness [63]. It is therefore possible that t(8;21) patients with AML with high serum Gal-3 levels may have unfavorable outcomes because of high CD56 expression in AML cells.

### 2.2. Gal-3 Is Highly Expressed by AML Cells and Synergizes with CD74/CD44 Signaling Pathway to Regulate AML Cell Survival

It is unclear how Gal-3 relates to possible Gal-3 target proteins. Ruvolo et al. used proteomic analysis to determine Gal-3 protein expression in blast cells derived from de novo patients with AML and to discover the protein networks linked to Gal-3. Furthermore, they investigated the prognostic impact of Gal-3 and its associated protein networks in these patients. The AML blast cells have significantly higher levels of Gal-3 protein than normal CD34^+^ cells. Gal-3 protein expression was highest in patients with AML with monocyte-containing FAB M4 and M5 subtypes. Additionally, there was a significant correlation between higher Gal-3 protein levels and lower blast percentages in the PB and higher monocyte percentages in the BM and PB. Patients with AML with higher Gal-3 protein levels had significantly shorter remission duration (RD) and were at a significantly higher risk of relapsing than those with lower Gal-3 protein levels. Furthermore, among patients who achieved remission, those with higher Gal-3 protein had a lower OS. This implies that following treatment, the protein is helping leukemic cells to recover and proliferate. Thirty-seven proteins positively correlated with Gal-3 protein expression levels in patients with AML, including the unmodified protein MAP2K1 (MEK1) and the phospho-proteins Protein Kinase B (AKT; pT308), Extracellular Receptor Kinase (ERK; pY202/pY204), Protein Kinase C (PKC) δ (pT507, pS645, and pS664) and PKC α (pS657), and SRC (pY416 and pY527) [64]. The PI3K/AKT/mTOR pathway is one of the most frequently activated pathways in patients with AML and is associated with drug resistance and poor prognosis. The PI3K-AKT-mTOR pathway is a critical signaling pathway for the growth, survival, proliferation, differentiation, and glucose metabolism of AML cells [65,66,67]. The MEK/ERK signaling pathway is frequently highly active in patients with AML [68,69] and contributes to clonal selection in AML, enabling immature CD34⁺ cells to survive and acquire resistance to chemotherapy [70]. It has been demonstrated that the activation of survival kinases, including AKT, ERK, and PKCα, predicts a poor clinical prognosis in patients with AML [71]. Active phosphorylated AKT (at S473) and active phosphorylated PKCδ (at T507 and S645) were positively correlated with phosphorylated glycogen synthase kinase GSK3α/β (S9/S21) protein expression levels in blast cells from de novo patients with AML, which reflects inactivation of the GSK3 kinase [72]. Research indicates that PKC may also have pro-survival characteristics [73,74,75]. Kinehara et al. suggested that PKC δ may phosphorylate GSK3 kinases in human pluripotent stem cells as part of a mechanism to regulate self-renewal [76]. However, since AKT was identified as a PKCδ kinase and GSK3 kinase, and PKCδ did not phosphorylate GSK3α/β in the AML cell lines, there seems to be no relationship between PKCδ and GSK3 in AML cells [72]. SRC-family kinases play important roles in cell proliferation, survival, and activation of oncogenic signaling pathways in AML [77]. SRC was found at significantly higher levels in the serum of de novo patients with AML compared to healthy individuals and negatively correlated with OS, which suggests that SRC may play a role in AML progression and poor prognosis [78]. Furthermore, MAP2K1 (MEK1) gene expression was higher in AML cells with increased LGALS3 expression according to the “The Cancer Genome Atlas” (TCGA) database [64]. The Gal-3 protein had a negative correlation with thirty-one other proteins, including the AKT protein phosphatases (members of the PP2A B subunit family, i.e., PPP2R2A/B/C/D). The inverse relationship between Gal-3 expression and PPP2R2A/B/C/D may indicate that Gal-3 suppresses PP2A [64]. The PPP2R2A-containing PP2A isoform dephosphorylates PKCα and AKT (at T308) [79,80]. Therefore, inhibition of the PP2A subunit by Gal-3 may lead to increased PKCα and AKT phosphorylation. Both the AKT and ERK signaling pathways are blocked when Gal-3 is suppressed by short hairpin RNA (shRNA) or an inhibitor of Gal-1 and Gal-3, named GCS-100 [81,82]. To determine if Gal-3 directly affects the different Gal-3 correlated proteins, Ruvolo et al. transduced an AML cell line with either LGALS3 shRNA or control shRNA. PP2A regulatory B subunits showed higher protein expression when LGALS3 was suppressed, which is in line with the proteomic analysis. Nevertheless, there was no significant change in PPP2R2A mRNA expression in the AML cell line because of LGALS3 suppression. Thus, LGALS3 was shown to directly regulate the members of the PP2A B subunit family in the AML cell line through a post-transcriptional process [64].

**Figure 2:**
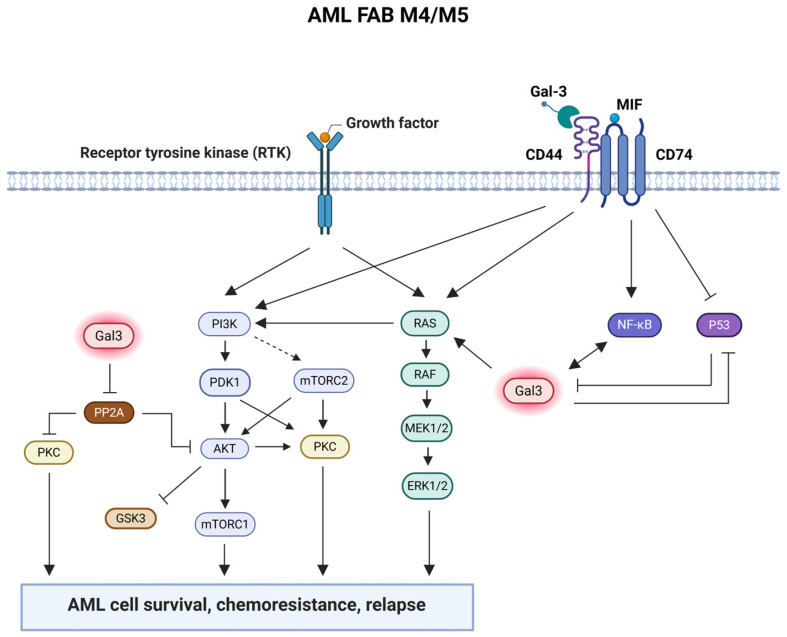
Gal-3 expression is high in blast cells from patients with AML with FAB M4/M5. Gal-3 suppresses the activity of PP2A and p53, and activates PKC, PI3K/AKT/mTOR, Ras/Raf/MEK/ERK, and NF-κB signaling pathways in AML cells, leading to cell survival, chemoresistance and relapse. The CD74/CD44 pathway can also promote AML cell survival by inhibiting p53 activation and activating the PI3K/AKT/mTOR, Ras/Raf/MEK/ERK, and NF-κB signaling pathways. NF-κB activation stimulates Gal-3 expression, whereas p53 activation inhibits it. MIF-activated CD74/CD44 signaling likely induces Gal-3 expression through NF-κB activation and p53 inactivation in AML cells. Gal-3 is also a ligand for CD44, the function of which in AML is unknown. Gal-3 and CD74 proteins regulate independent survival pathways, but when both pathways are activated, this may enhance the survival of AML cells and lead to the worst prognosis in patients with AML. Created with Biorender.com.

As a positive regulator of CD44 and CXCR4 signaling, the invariant chain protein CD74 [83,84,85] may be essential for AML cell survival and function [64]. Although CD74 was shown to bind the pro-inflammatory cytokine macrophage inhibitory factor (MIF), CD74 cannot start MIF signaling on its own; either the co-receptor proteins CD44 or CXCR4 are required [83,84,85,86,87]. MIF leads to CD74-dependent activation of the ERK, AKT, and JNK signaling pathways [88,89,90,91]. It has been demonstrated that CD74-dependent MIF signaling activates NF-κB and inhibits p53 function [91,92]. Galectin-3 activates NF-κB [93] and Gal-3 expression is associated with NF-κB activation [31]. Wildtype (wt) p53 inhibits the transcription of the LGALS3 gene, hence blocking its expression [94,95,96]. This data suggests that MIF-activated CD74/CD44 signaling induces LGALS3 expression by NF-κB activation and p53 inactivation in AML cells. Compared with normal CD34^+^ cells, AML blast cells also expressed more CD74 protein, which showed a strong positive correlation with CD44 protein expression. It is known that MIF promotes B cell survival by activating the CD74/CD44 receptor complex [91]. It is yet unclear how CD74 functions in AML blast cells. Next, protein networks based on Gal-3- and CD74-associated proteins were constructed using proteomic analysis. The Gal-3 protein network and the CD74 protein network were found to be strongly associated, where, among other things, the expression of Gal-3-positively correlated proteins and CD74/CD44 proteins were upregulated in patients with AML with both active Gal-3 and CD74 protein networks. However, the expression of CD74 and CD44 mRNA and protein did not change significantly after LGALS3 suppression in the AML cell line, suggesting that Gal-3 does not regulate CD74 and CD44 expression [64]. Metastasis is promoted by binding of Gal-3 to CD44 [97]. Furthermore, patients with AML with only active Gal-3 or CD74 protein networks had shorter OS and RD compared to normal state patients with AML, whereas patients with AML with both active protein networks were associated with the shortest OS and RD [64]. In conclusion, it is possible that Gal-3 and CD74 proteins regulate independent survival pathways, but when both pathways are activated, this may enhance the survival of AML cells and lead to the worst prognosis in patients with AML (Figure 2). This study implies that AML treatment may benefit from combined approaches targeting Gal-3 and CD74/CD44.

### 2.3. Gal-3 Expression in AML Cells Promotes Chemotherapeutic Resistance by Stimulating AML Cell Survival

**Figure 3:**
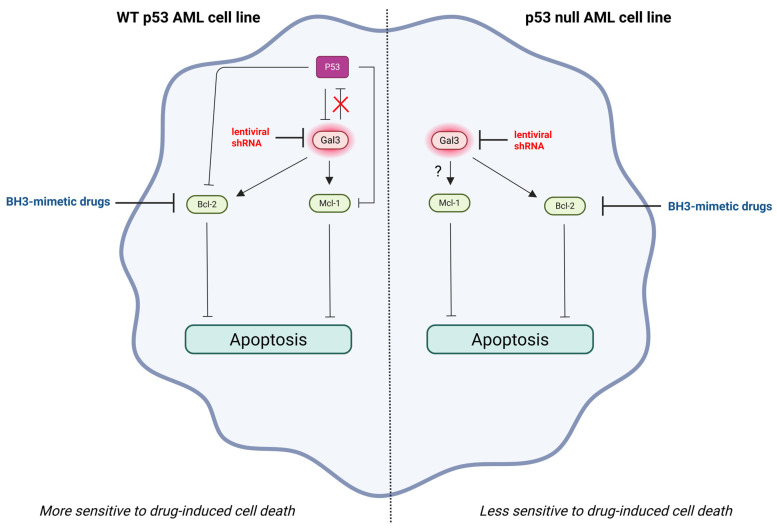
Gal-3 expression in AML cells promotes drug resistance. Silencing of Gal-3 by lentiviral LGALS3 shRNA renders the wt p53 AML cell line more sensitive to BH3 mimetic-induced apoptosis than the p53 null AML cell line. Silencing of Gal-3 reduces the expression of anti-apoptotic Mcl-1 protein in the wt p53 AML cell line, but increases the expression of Mcl-1 protein in the p53 null AML cell line due to the absence of the pro-apoptotic p53 protein in the p53 null AML cell line. The presence of p53 may contribute to drug-induced apoptosis in the absence of Gal-3. Active p53 reduces the expression of anti-apoptotic Mcl-1 and Bcl-2 proteins in the wt p53 AML cell line, either directly or via inhibition of Gal-3 expression. Gal-3 suppression in the p53 null AML cell line, only reduces Bcl-2 protein expression, suggesting that Gal-3 stimulates Bcl-2 protein expression independently of p53 regulation in this cell line. Created with Biorender.com.

The use of BH3 mimetics, which target the anti-apoptotic members of the B-cell lymphoma-2 (Bcl-2) family, may be problematic in the treatment of AML, since drug resistance may develop. Ruvolo et al. investigated the effect of Gal-3 suppression on BH3 mimetic-mediated AML cell killing. Therefore, Gal-3 was suppressed by lentiviral LGALS3 shRNA in two different AML cell lines, namely wt p53 OCI-AML3 and p53-null THP-1 cells, and subsequently treated with ABT-737, a BH3 mimetic agent targeting the anti-apoptotic proteins Bcl-XL and Bcl-2. Suppression of LGALS3 sensitized both AML cell lines to ABT-737, as evidenced by a decrease in viable cells and an increase in cell apoptosis. However, the AML cell line with wt p53 was substantially more sensitive to ABT-737-induced apoptosis after LGALS3 shRNA knockdown than the p53 null AML cell line. The increased sensitivity of the wt p53 AML cell line to ABT-737 could be due to differences in the amount of the pro-apoptotic protein p53 between both AML cell lines. Without a functional p53 pathway, cells are no longer forced to repair DNA damage or undergo apoptosis, giving them a survival advantage and allowing uncontrolled proliferation [98]. The presence of active p53 might aid in BH3 mimetic-mediated apoptosis in the absence of Gal-3. GCS-100 treatment induced p53 expression in the wt p53 AML cell line, which suggests that Gal-3 suppresses p53 function. To assess the role of p53 in drug-mediated apoptosis, p53 expression was knocked down with lentiviral p53 shRNA in the wt p53 AML cell line and then treated with ABT-737 with or without GCS100. The wt p53 AML cell line transduced with control shRNA was more sensitive to ABT-737 than the cell line transduced with p53 shRNA. Furthermore, the wt p53 AML cell line transduced with control shRNA showed synergistic killing with the combination treatment of GCS-100 and ABT-737. However, the wt p53 AML cell line expressing p53 shRNA was less sensitive to combination treatment with GCS-100 and ABT-737, suggesting that the presence of active p53 sensitizes the wt p53 AML cell line to GCS-100/BH3 mimetic-induced apoptotic cell death. Moreover, suppression of Gal-3 by LGALS3 shRNA reduced anti-apoptotic Mcl-1 protein expression in the wt p53 AML cell line, but increased Mcl-1 protein expression in the p53 null AML cell line. This finding suggests that the presence of wt p53 efficiently reduces Mcl-1 protein expression in the wt p53 AML cell line, which may clarify why suppression of LGALS3 had a stronger effect on ABT-737 sensitivity in the wt p53 AML cell line than in the p53 null AML cell line. Thus, Mcl-1 expression may be crucial for resistance to BH3 mimetics in the p53-null AML cell line [82]. The role of Mcl-1 expression in resistance to BH3 mimetics in many types of cancer has been described [99,100,101]. Mcl-1 is required for the development and sustained growth of AML [102]. Furthermore, Bcl-2 protein expression was reduced in both AML cell lines by LGALS3 suppression, which suggests that Gal-3 stimulates Bcl-2 protein expression in both AML cell lines [82]. It has been demonstrated that Gal-3 binds to Bcl-2 through a NWGR (Asp-Trp-Gly-Arg) motif shared by both proteins, assisting the anti-apoptotic molecule in maintaining mitochondrial membrane integrity under stress [103,104]. The negative regulation of Bcl-2 expression by the pro-apoptotic protein p53 has been reported [105], suggesting that p53 inhibits Bcl-2 protein expression either directly or via inhibition of Gal-3 expression in the wt p53 AML cell line. However, there may also be p53-independent regulation of Bcl-2 expression by Gal-3, as in the p53-null AML cell line (Figure 3).

Overall, these results suggest that Gal-3 expression in AML cells promotes drug resistance by stimulating AML cell survival. Thus, the use of potent Gal-3 inhibitors in combination with BH3 mimetics for the treatment of AML might significantly enhance their effectiveness. However, a serious side effect of ABT-737 is thrombocytopenia, as platelet homeostasis depends on Bcl-XL [82]. Venetoclax (ABT-199) is a selective BH3 mimetic drug that has high specificity for the Bcl-2 protein and triggers apoptosis in cancer cells. It is FDA approved for use in patients with chronic lymphocytic leukemia (CLL), small lymphocytic lymphoma (SLL), and newly diagnosed, elderly, or comorbid AML who are not candidates for intensive chemotherapy [106]. GCS-100 and ABT-199 alone were effective in reducing the viability of wt p53 AML and p53-null AML cell lines. However, when GCS-100 was combined with ABT-199 in both AML cell lines, it synergistically eliminated both AML cell lines, as a more than 99% reduction in viable cells was found. Since promising results were obtained with GCS-100 and venetoclax, it would be valuable to test venetoclax with specific Gal-3 inhibitors or Gal-3 shRNA in AML cells in the future.

### 2.4. BM-MSCs Induce Drug Resistance of AML Cells by Gal-3 Upregulation in AML Cells

The BM niche serves as a home for AML cells, which can result in chemoresistance and disease recurrence [3,4,107]. Previous studies have shown that stromal cells in BMM are important for the development and progression of leukemia [1,2]. Hu et al. investigated the function of Gal-3 in BMM-induced drug resistance of AML cells. The BMM was mimicked in vitro using human bone marrow-derived mesenchymal stromal cells (hBM-MSCs) obtained from normal donors. The effects of hBM-MSCs on drug resistance in an AML cell line and possible pathways for this were investigated, with an emphasis on the function of Gal-3 in the AML cell line. They demonstrated that hBM-MSCs dramatically reduced AML cell line apoptosis and increased the absolute number of surviving AML cell lines in response to cytotoxic drug exposure (Idarubicin, IDA). Thus, hBM-MSCs protect AML cells against death from cytotoxic drugs. They also discovered that both gene and protein expression levels of Gal-3 were significantly higher in the hBM-MSCs-conditioned AML cell line than in the unconditioned one, indicating that hBM-MSCs induce Gal-3 overexpression in AML cells. To clarify the precise function of hBM-MSC-induced Gal-3 overexpression in AML cell line drug resistance, they transfected the AML cell line with LGALS3 shRNA to silence Gal-3 expression. They showed that apoptosis of the Gal-3-silenced AML cell line increased in the presence of cytotoxic drugs, and that the protective effect of hBM-MSCs against cytotoxic drugs was diminished by Gal-3 silencing in the AML cell line [107]. By interacting with β-catenin, Gal-3 has been shown to be essential for Wnt signaling, which is linked to the enhancement of cell viability and cell cycle progression in pancreatic and colon cancer cells [108,109]. The development of leukemia is caused by the dysregulation of Wnt signaling, which is crucial for preserving normal hematopoiesis [110,111]. Wnt/β-catenin signaling is necessary for the development of AML [112,113]. Consequently, Hu et al. examined whether the Wnt/β-catenin signaling pathway plays a role in hBM-MSC-induced drug resistance of the AML cell line. Gal-3 overexpression in the AML cell line by hBM-MSCs enhanced β-catenin expression at the protein but not mRNA level in the AML cell line, which implies that Gal-3 overexpression stabilized β-catenin in the AML cell line, possibly by preventing its degradation. Silencing of Gal-3 by LGALS3 shRNA reduced β-catenin protein expression in the AML cell line when co-cultured with hBM-MSCs but not in the absence of hBM-MSCs. To better understand the role of Gal-3-induced β-catenin in AML cell line drug resistance, they treated the AML cell line with a specific Wnt/β-catenin signaling inhibitor ICG-001. They discovered that the Wnt/β-catenin signaling inhibitor significantly reduced the protective effect of hBM-MSCs against the apoptotic effects of cytotoxic drugs in the AML cell line [107]. Altogether, these results suggest that hBM-MSC-induced Gal-3 overexpression in the AML cell line stimulates β-catenin stabilization and activates the Wnt/β-catenin pathway in the AML cell line, which is critical for AML drug resistance. Expression of the Wnt/β-catenin pathway target genes, such as cyclin D1 [114] and c-Myc [115], was increased in the hBM-MSCs-conditioned AML cell line, and was downregulated after Gal-3 silencing in the hBM-MSCs-conditioned AML cell line [107]. By increasing the promoter activity of cyclin D1 through the transcription factor Sp1 and a cAMP-responsive element in human breast epithelial cells, Gal-3 has been shown to increase cyclin D1 gene expression [116]. Nevertheless, the expression of Wnt/β-catenin pathway target genes was not affected by Gal-3 silencing in the AML cell line cultured alone [107], which suggests that Gal-3 overexpression is needed to induce activation of the Wnt/β-catenin pathway and transcription of its target genes in the AML cell line. Therefore, it seems possible that some Gal-3 does not induce activation of the Wnt/β-catenin pathway and its associated genes in the AML cell line used in the absence of hBM-MSCs; this needs to be confirmed in other AML cell lines. Prior research has demonstrated that activation of PI3K/AKT signaling by Gal-3 increases GSK-3β phosphorylation (S9), which inactivates this serine/threonine kinase, leading to decreased β-catenin phosphorylation (S33, S37 and T41) and degradation, thereby stabilizing β-catenin and increasing its nuclear levels [108,109]. Hu et al. also observed that Gal-3 upregulation stimulated AKT and GSK-3β phosphorylation in the hBM-MSCs-conditioned AML cell line. β-catenin protein expression was upregulated in the AML cell line later than GSK-3β phosphorylation, indicating that Gal-3 stabilized β-catenin through GSK-3β phosphorylation in the hBM-MSCs-conditioned AML cell line. The overexpression of β-catenin protein in the hBM-MSCs-conditioned AML cell line was inhibited when the hBM-MSCs-conditioned AML cell line was treated with a PI3K/AKT signaling inhibitor LY294002, although Gal-3 protein expression remained unaffected [107]. Altogether, these findings showed that increased Gal-3 protein expression in the hBM-MSCs-conditioned AML cell line decreased β-catenin degradation by stimulating AKT and GSK-3β phosphorylation, leading to the enhanced transcription of β-catenin target genes, such as cyclin D1 and c-myc, hence promoting AML cell drug resistance. GSK-3β activation by inactivation of PI3K/AKT also causes Mcl-1 instability and subsequent mitochondrial apoptosis when growth factors are removed [117], implying that Gal-3 may also induce AML cell survival by causing Mcl-1 stabilization via PI3K/AKT activation and then GSK-3β inactivation.

Since Hu et al. demonstrated that Gal-3 upregulation is crucial in hBM-MSC-induced drug resistance of the AML cell line in vitro, they questioned whether this also applies to non-M3 patients with AML. Interestingly, the BM mononuclear cells from relapsed/refractory patients with AML expressed higher Gal-3 mRNA levels than those from primary patients with AML. Furthermore, hBM-MSCs induced the protein expression of Gal-3 and β-catenin in primary malignant cells derived from patients with AML [107]. This study also indicates that high Gal-3 plays a crucial role in the maintenance of MRD in addition to the development of drug resistance.

**Figure 4:**
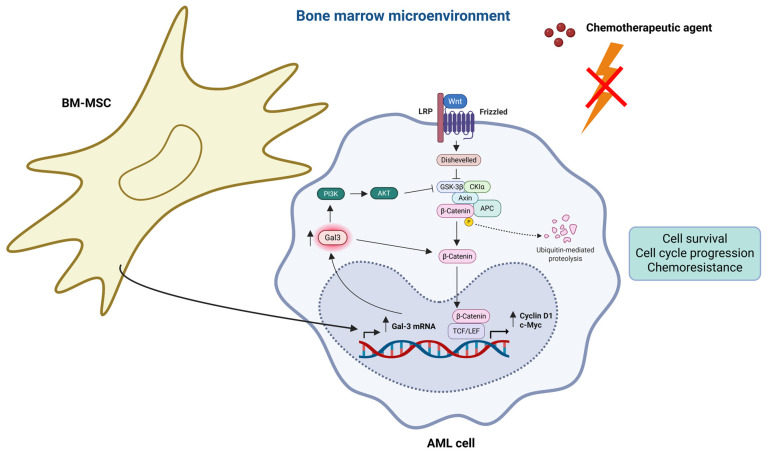
Role of Gal-3 overexpression in BM-MSC-induced drug resistance of AML cells. BM-MSCs promote AML cell survival, cell cycle progression, and chemoresistance by increasing both Gal-3 mRNA and protein expression in AML cells. Gal-3 overexpression promotes activation of Wnt signaling in AML cells. Elevated levels of Gal-3 bind to and increase the expression of β-catenin protein, implying that Gal-3 overexpression stabilizes β-catenin in AML cells by preventing its degradation. Gal-3 overexpression overactivates PI3K and AKT, which inactivates GSK-3β, leading to decreased β-catenin phosphorylation (S33, S37 and T41) and degradation. Overactive β-catenin translocates to the nucleus and increases transcription of β-catenin target genes, such as cyclin D1 and c-Myc. Created with Biorender.com.

Collectively, these results demonstrate that hBM-MSCs increase drug resistance of AML cells by upregulating Gal-3 protein expression in these cells. However, little is known about the way in which the BM niche interacts with these AML cells and the upstream pathways by which the BM niche cells induce Gal-3 overexpression in AML cells. Overall, hBM-MSC-induced Gal-3 overexpression in the AML cell line inhibits β-catenin degradation via overactivation of the PI3K/AKT pathway, which phosphorylates GSK-3β, thereby decreasing its activity. This leads to the stabilization and nuclear transport of β-catenin, whereafter it triggers the transcription of its downstream targets, which causes the AML cell line to become drug resistant. In patients with AML with intermediate cytogenetics, high levels of phosphorylated GSK3α/β (S9/S21) were associated with a shorter OS and RD. Furthermore, in the intermediate cytogenetic patients with AML, levels of both Gal-3 and phosphorylated AKT (S473) were positively correlated with phosphorylated GSK3α/β expression, indicating a function for Gal-3 in AKT-mediated repression of GSK3 in AML cells [72] (Figure 4). Thus, Gal-3 could be a possible therapeutic target in the treatment of BM-MSC-induced AML cell drug resistance.

### 2.5. AML Cell-Derived Extracellular Vesicles Upregulate Gal-3 Expression in BM-MSCs

It is thought that changes in the BMME contribute to the progression of leukemia. BM stromal cells, an essential component of the BMME, are remodeled by AML cells to provide a sanctuary for the progression of AML. Leukemic cells alter BM stromal cells to support their survival, proliferation, invasion, and drug resistance. There is increasing evidence that leukemic cells have a significant impact on BM stromal cells via their extracellular vesicles (EVs) that are secreted into the extracellular space. These are membrane-encapsulated nanoparticles [118,119]. EVs are then taken up by BM stromal cells, altering their function to promote leukemia growth. Currently, EVs are thought to be an extra means of communication between cells, enabling the interchange of genetic material, proteins, and lipids [118,120].

Kargar-sichani et al. examined the effect of EVs derived from the plasma of de novo non-M3 patients with AML on the molecular and cellular characteristics of normal BM-derived MSCs. They showed that AML patient-derived EVs increased the metabolic activity and viability of normal BM-MSCs in a concentration-dependent manner compared to normal EVs derived from healthy patients. They also found reduced reactive oxygen species (ROS) and Bax (pro-apoptotic) gene expression levels, and increased Ki-67 (nuclear cell proliferation marker) gene and protein levels and Bcl-2 (pro-survival) gene expression in normal BM-MSCs treated with AML-EVs. On the contrary, the highest concentration of AML-EVs was toxic to normal BM-MSCs as measured by reduced cell viability and induction of apoptosis, which was accompanied by a reduction of Bcl-2 gene expression and elevation of Bax gene expression [121]. This indicates that higher doses of AML-EVs cause normal BM-MSCs to undergo apoptosis, which may disrupt normal hematopoiesis and thus promote leukemogenesis and disease progression. Another interesting finding by Hu et al. was that hBM-MSCs obtained from normal donors also expressed Gal-3 [107]. Kargar-sichani found that gene expression of Gal-3 and IL-6 was significantly upregulated in AML-EV-treated normal BM-MSCs, while the highest concentration of AML-EVs had no significant effect on both gene expressions [121]. It has been shown that neuroblastoma cells stimulate IL-6 production in BM-MSCs by a Gal-3 dependent mechanism [122,123]

**Figure 5:**
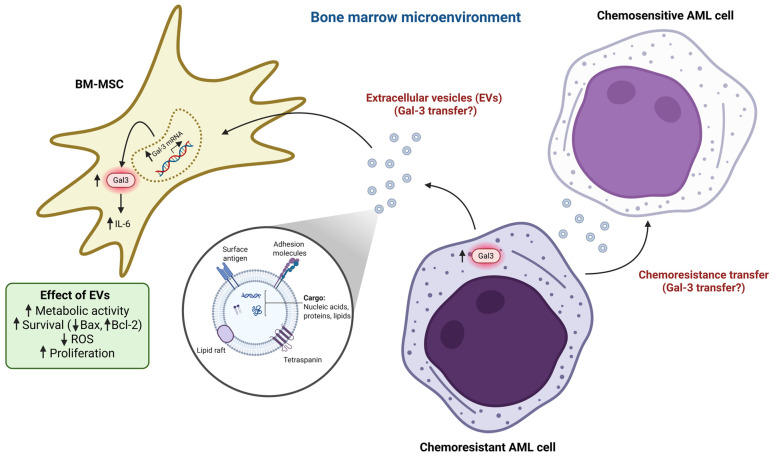
Chemoresistant AML cells can alter normal BM-MSCs and chemosensitive AML cells by secreting extracellular vesicles (EVs). These EVs are taken up by both cell types, which may be important in the development of chemoresistance. EVs derived from AML cells increase the metabolic activity, survival, and proliferation of BM-MSCs, while reducing oxidative stress in these cells. Furthermore, EVs derived from AML cells increase both Gal-3 and IL-6 gene expression in BM-MSCs. Whether the EV effects on BM-MSCs are caused by the upregulation of Gal-3 in BM-MSCs is still unknown. It is also unknown whether the EVs secreted by chemoresistant AML cells express Gal-3 to transfer it to the BM-MSCs and/or chemosensitive AML cells and/or activate a pathway leading to Gal-3 expression in these cells. Created with Biorender.com.

Altogether, these results indicate that AML-EVs increase the metabolic activity, survival, and proliferation of normal BM-MSCs when not present at high concentrations. The existence of BM-MSCs is also essential for leukemic cell survival [124]. It is not yet clear what the function is of the upregulation of Gal-3 in normal BM-MSCs by AML-EVs. Whether these effects are directly due to Gal-3 upregulation in normal BM-MSCs remains to be elucidated, by Gal-3 silencing in normal BM-MSCs in the presence of AML-EVs. Furthermore, it remains to be established how AML-EVs induce Gal-3 expression in normal BM-MSCs and what the paracrine effect of EVs is on nearby AML cells in terms of Gal-3 expression. Bouvy et al. have shown that EVs from chemoresistant AML cells interact with chemosensitive AML cells to transfer their chemoresistance to the chemosensitive AML cells [125]. Therefore, it is also interesting to elucidate whether chemoresistant AML cells transfer their Gal-3 via EVs to normal BM-MSCs and/or chemosensitive AML cells, which might be important in development of AML chemoresistance and relapse (Figure 5).

### 2.6. BM-MSCs Derived from AML Patients Highly Express Gal-3 Protein During Relapse

BM-MSCs significantly impact the AML patient outcomes. AML-derived BM-MSCs secrete various factors, which could impact the development and progression of AML [7,126]. Kornblau et al. compared the protein expression patterns of BM-MSCs derived from patients with AML at initial diagnosis with those derived from relapsed/refractory patients with AML by proteomic analysis and found differential expression of nine proteins. Among the nine proteins, Gal-3, phosphorylated β-catenin (p-CTNNB1; S33/S37/T41) and phosphorylated RPS6 were expressed at higher levels in BM-MSCs derived from relapsed/refractory patients with AML, while SMAD6, LYN, TCF4, integrin β3 (ITGB3), phosphorylated ELK1 and phosphorylated EIF4BP1 were higher in BM-MSCs derived from patients with AML at initial diagnosis [127]. Overall, these findings imply that high Gal-3 protein expression in BM-MSCs derived from patients with AML is associated with drug resistance and relapse. N-terminal phosphorylation of β-catenin at S33/S37/T41 designates β-catenin as a target for degradation, leading to low levels of cytoplasmic and nuclear β-catenin [128,129]. It is important to demonstrate this by measuring the β-catenin levels in the cytoplasmic and nuclear fractions in BM-MSCs derived from patients with relapsed/refractory AML.

### 2.7. BM-MSC-Derived Gal-3 Promotes AML Cell Adhesion and Survival

Understanding the biological pathways activated by Gal-3 in BM-MSCs and AML cells is important to develop more effective therapies against AML. Ruvolo et al. determined the biological pathways activated by Gal-3 in AML-derived MSCs. Firstly, they compared the Gal-3 protein expression levels between MSCs derived from the BM of healthy donors and de novo patients with AML by proteomic analysis, which was higher in de novo AML patient-derived MSCs. However, LGALS3 gene expression between AML-derived MSCs and healthy donor-derived MSCs was not significantly different, which indicates the involvement of a post-transcriptional or post-translational mechanism in the regulation of Gal-3 expression. Proteomic analysis was then used to correlate Gal-3 protein expression with other proteins in AML-derived MSCs, including variants of these proteins to identify biologically relevant pathways connected to Gal-3 in AML-derived MSCs. Gal-3 protein in AML-derived MSCs showed a positive correlation with the expression of thirteen proteins, including p-CTNNB1 (S33/S37/T41), Myc, CCND1 (cyclin D1), MAPK9, BAD, AKT2, CDK4, STAT1 and EGFR, while it negatively correlated with the expression of six proteins, including ITGB3, STMN1, LYN, and SIRT1. Interestingly, only the proteins that showed a positive correlation with Gal-3 were strongly interconnected [130]. Accordingly, this group previously identified that the PI3K/AKT signaling pathway was activated in AML-derived BM-MSCs. Furthermore, it was discovered before that BM-MSCs derived from relapsed/refractory patients with AML had lower expression levels of LYN and ITGB3 proteins, but higher expression levels of Gal-3 and p-CTNNB1 proteins [127]. The discovery that Gal-3 expression has a negative correlation with ITGB3 and LYN expression and a positive correlation with p-CTNNB1 expression in AML-derived MSCs, implies that these proteins may be part of a pathway that mediates MSC-induced drug resistance of AML cells. It has been reported that active WNT/β-catenin signaling supports stromal-mediated protection of AML cells [113,131,132,133]. Though a role for Gal-3 as a positive regulator of β-catenin is well recognized [107,108,109,134,135,136], it is currently unclear how Gal-3 might induce the transcription of β-catenin target genes, such as Myc and cyclin D1 in BM-MSCs from patients with AML, since β-catenin was phosphorylated at S33/S37/T41. This implies that alternative pathways for the transcription of β-catenin target genes in BM-MSCs from patients with AML may exist. Kornblau et al. found higher protein expression of phosphorylated epidermal growth factor receptor (p-EGFR, Tyrosine/Y992) in AML-derived BM-MSCs compared to normal BM-MSCs, which is highly associated with the PI3K/AKT pathway [127]. EGFR-induced activation of the PI3K/AKT pathway plays a crucial role in G1/S cell cycle progression by the induction of cyclin D1 activity and expression [137]. AKT activation in response to EGFR signaling can also phosphorylate β-catenin at S552 in vitro and in vivo, which leads to its dissociation from cell–cell contacts and translocation into the nucleus, thereby increasing its transcriptional activity and tumor cell invasion [138]. Phosphorylation of several sites at the C-terminus of β-catenin [at S552 by AKT, at S675 by PKA, and at S191/605 by JNK2 (MAPK9)] stabilizes β-catenin, leading to its nuclear translocation and activation of target genes [129]. Consistent with the proteomic analysis, LGALS3 suppression by lentiviral shRNA in normal BM-MSCs resulted in decreased protein expression of Myc, AKT and phosphorylated AKT (at S473), while ITGB3 protein expression was induced [130]. These results suggest that Gal-3 impairs ITGB3 expression and induces the expression of AKT and Myc, and AKT activity in BM-MSCs. A limitation of this experiment was that the effect of Gal-3 inhibition on Gal-3 correlated proteins was investigated in normal BM-MSCs rather than AML patient-derived BM-MSCs. Furthermore, it would be interesting to evaluate the effect of LGALS3 suppression in AML patient-derived BM-MSCs on other Gal-3 correlated proteins (listed in ref [130]). It is also important to show the CTNNB1 levels in the cytoplasmic and nuclear subcellular fractions to prove that β-catenin is translocated to the nucleus in AML-derived BM-MSCs.

Interestingly, transforming growth factor beta (TGF-β) was identified as an upstream regulator of Gal-3 protein and the proteins that positively correlated with Gal-3 in AML-MSCs [130], which is a cytokine secreted by MSCs within the BM niche. Blockade of TGF-β1 promotes primary AML cell chemosensibility to cytarabine when co-cultured with normal BM-MSCs [139]. The protective effect of BM-MSCs is reversed when TGF-β1 is blocked in co-cultures of AML cell lines and normal BM-MSCs [140], indicating that TGF-β plays a role in MSC-induced AML cell survival and chemoresistance. TGF-β1-induced AKT/β-catenin activation is mediated by Gal-3 [141,142]. The fate, phenotype, and function of AML-derived BM-MSCs can be impacted by Gal-3. Kornblau et al. identified that AML patient-derived BM-MSCs are more senescent than normal BM-MSCs [127], which might be due to the overexpression of Gal-3 protein by TGF-β induction in AML patient-derived MSCs, as senescent MSCs express and secrete high levels of TGF-β [143] and Gal-3 [144]. However, we cannot exclude the role of other proteins in the senescence of AML-derived BM-MSCs. Kornblau et al. showed higher protein expression of STAT1 in AML-derived BM-MSCs compared to normal BM-MSCs, which is highly associated with the PI3K/AKT pathway [127] and positively correlated with Gal-3 protein in AML-MSCs [130]. The interplay of PI3Kα and STAT1 enhances the anti-inflammatory MSC2 phenotype and plays a crucial role in IFN-γ-induced indoleamine 2,3-dioxygenase (IDO) production in MSC2, which leads to suppression of T cell proliferation. PI3Kα pathway activation is needed for full IFN-γ-induced STAT1 activation by mediating STAT1 phosphorylation (S727) [145]. The differentiation potential of AML-MSCs is different from normal BM-MSCs, which may be affected by AML cells, as the AML cell line induces osteogenic differentiation of normal BM-MSCs. AML-MSCs are directed toward osteogenic differentiation, whereas normal BM-MSCs differentiate into adipocytes [7,127,146]. Diffuse idiopathic skeletal hyperostosis (DISH)-derived BM-MSCs have a stronger osteogenic differentiation capacity than normal BM-MSCs, which may be due to the increased secretion of Gal-3 by DISH-BMSCs that activates the Wnt/β-catenin signaling by increasing β-catenin protein expression and its nuclear accumulation [147]. S-96 phosphorylation of Gal-3 is involved in osteogenic differentiation by enhancing intracellular β-catenin protein levels [148]. It has been demonstrated that overexpression of microRNA-21 (miR-21) stimulated the osteogenic differentiation of human umbilical cord mesenchymal stem cells (hUMSCs) by activating the PI3K/AKT pathway, leading to the stabilization and nuclear translocation of β-catenin to induce the expression of osteogenesis-related genes [149]. AKT inhibits the pro-apoptotic protein BAD by phosphorylating at S136, which inactivates this protein, promoting cell survival [150]. Furthermore, Gal-3 induces BM-MSC migration via AKT phosphorylation at S473 [151]. Myc plays a crucial role in MSC proliferation, especially under hypoxic conditions [152]. Gal-3 plays an essential role in MSC survival, migration, and proliferation [153]. Therefore, it seems possible that Gal-3 might also stimulate the survival, migration, and proliferation of AML-derived BM-MSCs.

**Figure 6:**
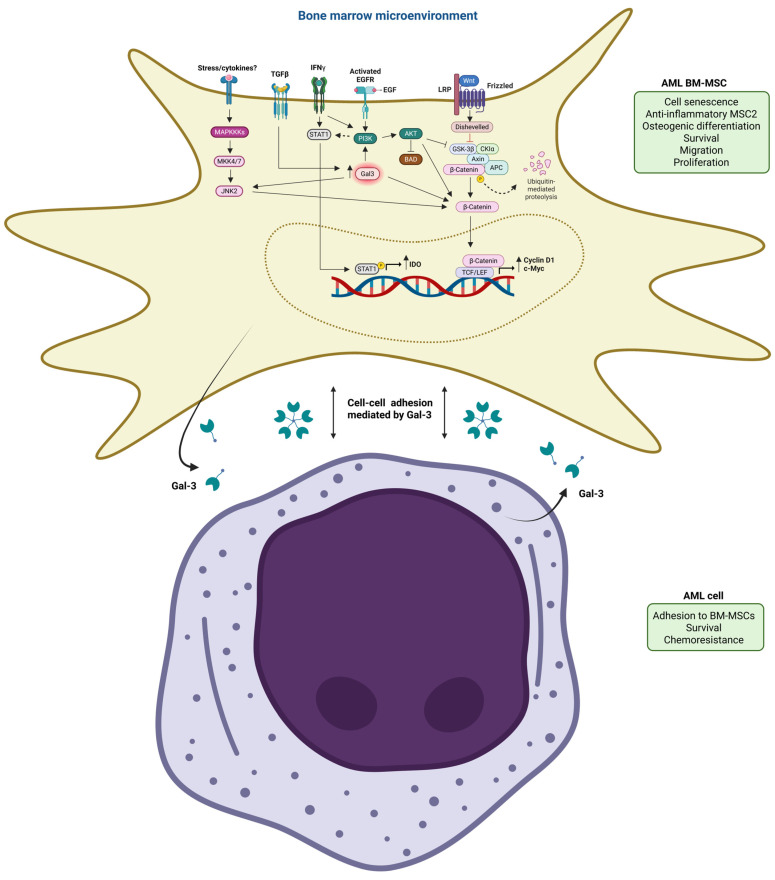
High Gal-3 protein expression in BM-MSCs derived from patients with AML can induce AML cell adhesion to BM-MSCs, AML cell survival and chemoresistance. Overexpression of Gal-3 protein in MSCs derived from patients with AML may be associated with cell senescence, anti-inflammatory MSC2 phenotype, osteogenic differentiation, cell survival, migration, and proliferation. TGF-β is an upstream regulator of Gal-3 protein. High Gal-3 expression in BM-MSCs derived from patients with AML overactivates, JAK/STAT, JNK, PI3K/AKT and Wnt/β-catenin signaling. Activation of JAK/STAT signaling by IFN-γ plays a crucial role in the production of indoleamine 2,3-dioxygenase (IDO) and thereby promotes the MSC2 phenotype. Activation of EGFR also leads to activation of the PI3K/AKT pathway. While AKT inhibits the pro-apoptotic protein BAD, it also stabilizes β-catenin together with JNK2. Intact β-catenin translocates to the nucleus, where it increases transcription of β-catenin target genes, such as c-Myc and cyclin D1, in BM-MSCs from patients with AML. Created with Biorender.com.

Ruvolo et al. also determined the effect of Gal-3 derived from MSCs on AML cells. The addition of a Gal-3 inhibitor CBP.001 (Carbohydrate Binding Protein version 001) to the co-culture of an AML cell line with normal BM-MSCs significantly decreased the number of viable AML cell lines by inducing apoptosis, and enhanced cytarabine-induced apoptosis of the AML cell line. These findings showed that Gal-3 inhibition might enhance chemotherapy-induced AML cell death in the presence of normal BM-MSCs. However, a limitation of this study was the use of normal BM-MSCs instead of BM-MSCs derived from patients with AML, as the latter are known to express higher levels of Gal-3 protein. Then, the effect of Gal-3 knockdown in normal BM-MSCs by lentiviral LGALS3 shRNA on the adhesion of an AML cell line to MSCs was examined. The number of AML cell lines that adhered to MSC LGALS3 shRNA transductants was diminished compared to MSC control shRNA transductants. This finding suggests that Gal-3 derived from normal BM-MSCs induces AML cell adhesion to MSCs, which also needs to be evaluated with BM-MSCs derived from patients with AML. It would also be interesting to evaluate the effect of Gal-3 knockdown in AML patient-derived BM-MSCs and relapsed/refractory AML patient-derived BM-MSCs on AML cell survival, proliferation, and migration. In summary, MSC-derived Gal-3 is crucial for both AML cell survival and adherence to MSCs, indicating that Gal-3 mediates MSC-induced drug resistance of AML cells [130] (Figure 6). Thus, Gal-3 targeting may be an effective microenvironment-based approach to treat patients with AML, especially patients with relapsed/refractory AML.

## 3. Therapeutic Targeting of Gal-3: Insights Gained from Other Diseases

Although most of the data on Gal-3 have been obtained from in vitro studies of AML, the role of Gal-3 in AML has not been confirmed in relevant animal models of AML. Given its ability to participate in multiple biological processes during AML chemoresistance, the idea that it could serve as a molecular target has been strongly raised. Several attempts to block Gal-3 expression with lentiviral shRNA or its function with a carbohydrate-derived competitive inhibitor have so far yielded encouraging results in experimental in vitro models of AML. However, animal studies have not been conducted to evaluate the potential of Gal-3 as a therapeutic target in AML chemoresistance. In this context, Gal-3 inhibitors have been tested in other diseases, opening the way for anti-Gal-3 therapy and increasing the potential of this protein as a target in AML chemoresistance and relapse. Natural compounds (polysaccharide-based inhibitors) and potent synthetic small-molecule inhibitors are examples of Gal-3-targeted therapeutics that have been tested in preclinical studies or clinical trials for several human diseases [18,154]. The small-molecule inhibitors, such as GB0139 (inhalable; formerly known as TD139) and GB1211 (oral; also known as selvigaltin), have higher selectivity and affinity (at nanomolar levels) for Gal-3 compared to large-molecule inhibitors, such as pectin derivatives (i.e., GCS-100, GM-CT-01, GR-MD-02) [18,155]. Therefore, in this review, inhibitors GB0139 and GB1211 were evaluated primarily for their ability to inhibit Gal-3-related processes.

TD139 has been shown to effectively inhibit the binding and endocytosis of Gal-3 in a carbohydrate-dependent manner in non-macrophage cells and M2 macrophages in vitro [156]. TD139 has shown positive results in the treatment of idiopathic pulmonary fibrosis (IPF). TD139 was found to be safe and well tolerated in a phase 1/2a clinical study (NCT02257177) of healthy volunteers and IPF patients and was observed to reduce Gal-3 expression in alveolar macrophages and plasma biomarkers associated with IPF progression, e.g., platelet-derived growth factor (PDGF)-BB, plasminogen activator inhibitor (PAI)-1, Gal-3, chemokine (C-C motif) ligand (CCL)-18 and YKL-40 [157]. TD139 has also demonstrated antifibrotic activity in murine models of lung fibrosis. TD139 inhibited TGF-β1-induced β-catenin activation in vitro and in vivo and diminished bleomycin-induced lung fibrosis in vivo [141]. GB0139 treatment can reduce the progression of acute lung injury (ALI) in vivo by reducing the recruitment and activation of inflammatory cells, the activation of pro-inflammatory M1 macrophages, pro-inflammatory cytokines (e.g., IL-6 and TNF-α) and pro-fibrotic cytokines (e.g., MIP-1α) in bronchoalveolar lavage fluid (BALf), while increasing cytotoxic CD3^+^CD8^+^ T cells. GB0139 inhibits inflammation in vitro by reducing neutrophil activation, monocyte IL-8 secretion, pro-inflammatory M1 macrophage activation, T cell apoptosis, and pro-inflammatory genes (e.g., IL-6, IL-8, TNF-α) in injured alveolar epithelial cells [158]. Furthermore, TD139 inhibits corneal angiogenesis and fibrosis in vivo and reduces VEGF-A-induced endothelial cell migration and sprouting in vitro [159].

GB1211 inhibits cell surface Gal-3 expression in human macrophages and TGF-β-induced pro-fibrotic gene expression in human hepatic stellate cells in vitro. Furthermore, this inhibitor exhibits anti-fibrotic activity in carbon tetrachloride (CCl_4_)-induced liver fibrosis and bleomycin-induced lung fibrosis mouse models [155]. Comeglio et al. examined the effectiveness of GB1211 in a high-fat diet (HFD) rabbit model of metabolic-associated steatohepatitis (MASH). They found that GB1211 decreased Gal-3 mRNA and protein levels in the liver. Furthermore, it reduced liver inflammation (e.g., infiltrates of macrophages), fibrosis (e.g., collagen deposition), biomarkers of liver disease (AST, ALT, bilirubin), and markers of inflammation and fibrosis at mRNA and protein levels [e.g., IL6, collagen, TGF-β3, snail family transcriptional repressor 2 (SNAI2)] [160]. Aslanis et al. evaluated the relative bioavailability, pharmacokinetic parameters, and safety of the tablet formulation of selvigaltin in a phase 1 clinical trial with healthy volunteers (GALBA-1; NCT05747573). Under fasting conditions, the tablet form of selvigaltin has been shown to have higher bioavailability than the capsule form by achieving higher plasma concentrations and higher systemic exposure, potentially offering a more effective treatment. Furthermore, food intake had minimal effects on the pharmacokinetics of the tablet form. It was stated that the tablet form offers a more convenient administration option for the patients. In addition, the 100 mg tablet form of selvigaltin was reported to be well tolerated in healthy volunteers and did not cause serious side effects. This study also suggests that further clinical studies of the tablet form should be conducted without specific food restrictions to evaluate its anti-fibrotic and anti-tumor activities [161]. It has been reported that Gal-3 enhanced the protein expression of immune checkpoint ligand PD-L1 in lung cancer cells in vitro by increasing STAT3 phosphorylation, while a Gal-3 inhibitor (GB1107) decreased both PD-L1 expression and STAT3 phosphorylation in these cells. The Gal-3 inhibitor enhanced the cytotoxic activity of T cells against lung cancer cells induced by PD-L1 blockade in vitro. In a mouse xenograft model of lung cancer, the combination of PD-L1 blockade and Gal-3 inhibitor synergistically reduced tumor growth, which was accompanied by increased infiltration of CD3^+^ tumor-infiltrating lymphocytes (TILs) and granzyme B release into tumors [162]. Another study found that Gal-3 helps cancer cells evade host immune surveillance by binding to immune checkpoint molecules. They showed that Gal-3 promotes PD-1/PD-L1 binding and reduces the binding of immune checkpoint inhibitors atezolizumab and pembrolizumab to PD-L1 and PD-1, respectively. GB1211 reduced the binding of Gal-3 to both PD-1 and PD-L1, and restored the binding of atezolizumab and pembrolizumab to PD-L1 and PD-1, respectively. The combination treatment with GB1211 and a blocking anti-PD-L1 monoclonal antibody reduced tumor growth in an LLC1 syngeneic mouse lung cancer model, which was accompanied by an increased percentage of CD8^+^ TILs [163] (Table 1). Overall, studies show that GB1211 enhances the response to immune checkpoint inhibitors. This suggests that Gal-3-targeted therapies can be used as a combination therapy with immune checkpoint inhibitors and may be more effective than monotherapy, highlighting the potential rationale for combination therapy also in AML. A phase 1/2 clinical trial (GALLANT-1; NCT05240131) is currently underway assessing the safety and efficacy of selvigaltin in combination with atezolizumab (anti-PD-L1 monoclonal antibody) versus placebo plus atezolizumab in patients with non-small cell lung cancer (NSCLC). Furthermore, a phase 2 clinical study (NCT05913388) investigates the objective response of selvigaltin and pembrolizumab (Keytruda; anti-PD-1 monoclonal antibody) versus placebo and pembrolizumab in patients with metastatic melanoma or head and neck squamous cell carcinoma (HNSCC). The results of both studies will provide further insight into the applicability of selvigaltin as a combinatorial treatment with standard immune checkpoint inhibitors.

## 4. Future Directions

There are currently no preclinical and clinical studies evaluating the effect of potent synthetic small-molecule Gal-3 inhibitors in AML. Consequently, clinical trials are needed to test the safety and efficacy of these compounds in relapsed/refractory patients with AML, either alone or in combination with other therapies. However, in vitro studies in human cells and in vivo studies in animals should be conducted with these inhibitors before clinical testing of these inhibitors. The use of potent Gal-3 inhibitors may lead to improved clinical outcomes in high-risk patients with AML with poor prognosis. This may provide an important treatment option for this patient group in the future, either as an add-on to existing/new therapies or as a stand-alone treatment. However, developing more specific and effective formulations of these synthetic small-molecule Gal-3 inhibitors may lead to more successful results in the future.

Although some of the signaling pathways involving Gal-3 have been identified, the precise mechanisms of its action and regulation in AML chemoresistance and relapse are not yet clear and require further mechanistic studies. The cell surface receptors and intracellular molecules involved in Gal-3 binding, and regulation of Gal-3 expression and function in both AML cells and BM-MSCs need to be well established. It is known that Gal-3 interacts with several β-galactoside-containing cell surface ligands, such as MUC-1 [97,165,166,167], TGF-βR [168], EGFR [168], VEGFR2 [169], PDGFRβ [170], Mac-2 binding protein [171,172,173], N-cadherin [174], αvβ3 integrin [175,176], CD45 [177], CD44 [178], β1 integrin [178,179], CD66 [180], LAMP [180], CD98 [181], CD147 [179], and TLR4 [182]. Among other intracellular ligands of Gal-3, Alix [183], nucling [184], synexin [33] and Sp1 [185] are known, in addition to K-RAS, β-catenin, and Bcl-2. However, the effect of Gal-3 interactions with these ligands in AML is unknown. In addition to TGF-β, NF-κB and p53 as described in this review, other proteins may also regulate Gal-3 expression. Overexpression of miR-152 in glioblastoma stem cells reduced cell proliferation, migration, and invasion, but enhanced apoptosis by downregulating Krüppel-like factor 4 (KLF4) protein expression, which leads to inhibition of Gal-3 expression and MEK1/2 and PI3K signaling. KLF4 normally binds and activates the Gal-3 promoter in glioblastoma stem cells. Moreover, miR-152 overexpression and KLF4 knockdown resulted in the smallest tumor volume and longest survival in nude mice [186]. Kim et al. have shown that conditional overexpression of Tim-3 in lung-derived myeloid cells causes lung inflammation in mice by increasing Gal-3 expression and secretion. However, when Tim-3 was blocked or the Gal-3 inhibitor GB1107 was administered in myeloid cell-specific Tim-3 knock-in mice, lung inflammation was reduced. Using an LPS-induced lung inflammation model with myeloid cell-specific Tim-3 knockout mice, they confirmed the association of Tim-3 with both Gal-3 expression and lung inflammation [164]. Since AML cells also overexpress Tim-3 [187], it is possible that Gal-3 expression and secretion are induced by enhanced Gal-9/Tim-3 signaling in AML cells, which remains to be confirmed. Regarding the regulation of Gal-3 function, posttranslational modification of Gal-3, such as phosphorylation at S6 by casein kinase 1 (CK1) induces Gal-3 export from the nucleus to the cytoplasm and protects the human breast carcinoma cell line from chemotherapeutic drug-induced apoptosis by activating JNK2 [36]. A polymorphism in the Gal-3 gene at position 191 (rs4644) where proline is replaced by histidine (P64H) confers sensitivity to MMP cleavage and the development of resistance to drug-induced apoptosis. The H/H homozygous genotype is associated with an increased risk of breast cancer in both Caucasian and Asian women [188]. It will be interesting to find out whether this nsSNP is also linked to an increased risk of developing drug resistance and disease relapse in patients with AML.

Another area worth investigating is how Gal-3 regulates the function of BM-MSCs and the interaction of AML cells with BM-MSCs, other cells in the BMME, and the ECM. Gal-3 overexpression in human breast carcinoma cell lines increases their adhesion to vitronectin, fibronectin, and laminin, which protects them from apoptosis [189]. How upregulation of Gal-3 in the AML BMME modulates immune cells has not been studied. A plausible hypothesis is that Gal-3 secreted from AML cells and BM-MSCs may promote immune escape by interacting with T cells, inhibiting their function, and inducing their apoptosis [190]. Extracellular Gal-3 induces apoptosis of human T cell lines through Gal-3 binding to CD45 and CD71 and clustering of CD71 on the T cell surface [191]. Gal-3 induces both phosphatidylserine (PS) exposure and apoptosis in primary activated human T cells [192]. Gal-3 detachment with an anti-Gal-3 monoclonal antibody improves the function of human CD8^+^ TILs by increasing IFN-γ secretion upon ex vivo restimulation. Gal-3 detachment likely promotes TCR/CD8 colocalization in these cells [193]. Gal-3 also interacts with LAG-3, which is needed for Gal-3-mediated suppression of IFN-γ production by CD8^+^ T cells in vitro. Gal-3 depletion leads to increased CD8^+^ T-cell effector function and tumor-free survival in vivo [194]. Gal-3 knockdown in BM-MSCs decreased the MSC inhibitory effect on T-cell proliferation [195]. Gal-3 also suppresses immune surveillance mediated by human NK cells. Gal-3 released from human tumor cells interacts with natural killer protein 30 (NKp30) on the surface of human NK cells in vitro, thereby inhibiting NK-cell-mediated cytotoxicity. Moreover, lentiviral knockdown of Gal-3 in human tumor cells leads to increased sensitivity to lysis by NK cells, while Gal-3-overexpressing human tumor cells are less sensitive to NK cell killing. Injection of NOD-SCID mice with Gal-3-silenced human tumor cells or Gal-3-overexpressing human tumor cells, followed by intratumoral injection of human NK cells, resulted in a decrease or increase in tumor volume, respectively, compared with injection with untreated tumor cells. This study indicates that Gal-3 suppresses human NK cell attack on tumors in vivo [196]. The NK-activating receptor natural killer group 2 member D (NKG2D) is important for tumor rejection by human NK cells after binding its tumor-associated ligand, major histocompatibility complex class I-related chain A (MICA), which is expressed on the human bladder tumor cell surface. When Gal-3 binds to the NKG2D-binding site of MICA via the core2 O-glycans of MICA, the recognition of metastatic human bladder tumor cells by human NK cells is impaired, and the activation of NK cells is severely suppressed by a strong reduction in the secretion of IFN-γ and granzyme B [197]. Binding of Gal-3 to another ligand of the NK cell receptor, MUC-1 via the core2 O-glycans of MUC-1 impairs the interaction of metastatic human bladder tumor cells with human NK cells, leading to escape from NK cell cytotoxicity [198].

It is also unknown whether upregulation of Gal-3 in BMME promotes neo-angiogenesis and subsequent infiltration of AML cells into extramedullary sites, which could be investigated. Gal-3 inhibition by a small-molecule Gal-3 inhibitor inhibits corneal angiogenesis and fibrosis in vivo and reduces VEGF-A-induced endothelial cell migration and sprouting in vitro [159]. High expression and secretion of Gal-3 from the tumor induce M2-like macrophage infiltration, which leads to enhanced tumor angiogenesis and tumor growth in vivo [199]. Cleavage of extracellular Gal-3 by MMPs is crucial for tumor angiogenesis and tumor growth in vivo [200]. Gal-3 promotes the migration and invasion of human pancreatic cancer cells and tongue cancer cells in vitro by increasing Akt phosphorylation and β-catenin transcriptional activity, leading to enhanced MMP-2 and -9 mRNA and protein expression [109,201]. High BM Gal-3 expression and plasma Gal-3 levels were found in patients with AML with FAB M4/M5 subtypes [8,45], which have extramedullary relapse [202,203,204]. High BM Gal-3 expression was also found in non-M3 patients with AML with PTPN11 mutation [8], which occurred more frequently in patients with AML with FAB M4/M5 subtypes [49] and patients with AML with extramedullary manifestations [205]. T(8;21) patients with AML with high serum Gal-3 levels may have unfavorable outcomes because of high CD56 expression in AML cells [46], which has been associated with extramedullary relapse [202,206].

## 5. Conclusions

AML remains a fatal disease despite conventional chemotherapy and stem cell transplantation. Numerous new strategies for the treatment of AML have emerged, which are under investigation [187,207,208,209,210]. Many studies to date have clearly demonstrated that Gal-3 is a useful biomarker for determining poor prognosis in patients with AML. There is emerging evidence that high Gal-3 expression plays a role in AML chemoresistance and relapse, and may exert leukemia-promoting activities by interacting with various molecules and activating various signaling pathways. Examples of activated pathways include PI3K/AKT/mTOR, Ras/Raf/MEK/ERK, JAK/STAT, JNK, Wnt/β-catenin, PLC/PKC and NF-κB, which are interconnected to promote AML cell survival and chemoresistance. Gal-3 is a pro-survival protein that, when highly expressed in AML cells, protects these cells from chemotherapeutic drug-induced apoptosis. BMME’s contribution to AML progression is gaining increasing attention. BM-MSCs promote AML cells to acquire drug resistance by inducing Gal-3 overexpression in AML cells. Research has also shown that changes in BM-MSCs induced by AML cells can drive the progression of AML. In this regard, BM-MSCs can be manipulated by EVs secreted from AML cells to upregulate Gal-3 expression in BM-MSCs. It will be interesting to discover whether the EVs secreted by these AML cells express Gal-3 to transfer it to these BM-MSCs and/or activate a pathway leading to Gal-3 expression in BM-MSCs. The transfer of exosomal Gal-3 protein from stromal cells into human ALL cells, leading to NF-κB activation and to its own transcription in human ALL cells, has been described by Fei et al. [10]. Gal-3-overexpressing BM-MSCs can be senescent, anti-inflammatory, viable, migratory, proliferative, and undergo osteogenic differentiation. BM-MSC-secreted Gal-3 further promotes AML cell adhesion and survival. Overall, AML cells alter the function of BM-MSCs through Gal-3 upregulation in BM-MSCs, and Gal-3 upregulation in the BMME provides survival signals to AML cells, thereby creating a self-reinforcing niche conducive to escape from chemotherapy and the immune response.

Taken together, these findings indicate that there is a bidirectional interaction between AML cells and BM-MSCs, mediated by Gal-3, which contributes to the progression of AML. Currently, there is no effective treatment for relapsed/refractory patients with AML. Downregulation of Gal-3 expression or activity may be a promising therapeutic strategy to restore the BM niche, enhance the response of AML cells to conventional chemotherapeutic drugs, and improve patient outcomes. Potent synthetic small-molecule Gal-3 inhibitors offer a promising approach for the treatment of relapsed/refractory AML, although more research is needed. However, both preclinical and clinical studies support their efficacy and potential benefits in several diseases.

## Figures and Tables

**Table 1:** Effects of Galectin-3 inhibitors in disease models.

Disease Model/Cell Type	Drug	Effects	Study Type	Reference
THP-1 (M0, M1, M2), HFL-1, SKBR3 cell lines	TD139	Binding and endocytosis of Gal-3 is inhibited in a carbohydrate dependent manner in non-macrophage cells and M2 macrophages.	Preclinical	[156]
Healthy subjects and IPF patients	TD139	TD139 was safe and well tolerated in healthy subjects and IPF patients. TD139 reduced Gal-3 expression in alveolar macrophages and plasma biomarkers associated with IPF progression (PDGF-BB, PAI-1, Gal-3, CCL-18, and YKL-40).	Phase 1/2a clinical trial (NCT02257177)	[157]
Primary alveolar epithelial cells from WT mice (in vitro), mouse model of TGF-β1-induced lung fibrosis (in vivo)	TD139	TD139 inhibited TGF-β1-induced β-catenin activation in vitro and in vivo.	Preclinical	[141]
Mouse model of bleomycin-induced lung fibrosis	TD139	TD139 diminished lung fibrosis in vivo.	Preclinical	[141]
Peripheral human neutrophils and monocytes, and cell lines: THP-1, Jurkat E6, A549	GB0139	GB0139 inhibits inflammation in vitro by reducing neutrophil activation, monocyte IL-8 secretion, pro-inflammatory M1 macrophage activation, T cell apoptosis, and pro-inflammatory genes (e.g., IL-6, IL-8, TNF-α) in injured alveolar epithelial cells.	Preclinical	[158]
Mouse model of LPS/bleomycin-induced ALI	GB0139	GB0139 reduces the progression of ALI in vivo by reducing the recruitment and activation of inflammatory cells, the activation of pro-inflammatory M1 macrophages, pro-inflammatory cytokines (e.g., IL-6 and TNF-α) and pro-fibrotic cytokines (e.g., MIP-1α) in BALf, while increasing cytotoxic CD3^+^CD8^+^ T cells.	Preclinical	[158]
Human umbilical vein endothelial cells (HUVECs)	TD139	TD139 reduces VEGF-A-induced endothelial cell migration and sprouting in vitro.	Preclinical	[159]
Mouse models of silver nitrate cautery and alkaline burn injury	TD139	TD139 inhibits corneal angiogenesis and fibrosis in vivo.	Preclinical	[159]
THP-1, LX2 cell lines	GB1211	GB1211 inhibits cell surface Gal-3 expression in human macrophages and TGF-β-induced pro-fibrotic gene expression in human hepatic stellate cells in vitro.	Preclinical	[155]
Mouse models of CCl4-induced liver fibrosis and bleomycin-induced lung fibrosis	GB1211	GB1211 exhibits anti-fibrotic activity in the liver and lungs in vivo.	Preclinical	[155]
HFD rabbit model of MASH	GB1211	GB1211 decreased Gal-3 mRNA and protein levels in the liver. GB1211 reduced liver inflammation (e.g., infiltrates of macrophages), fibrosis (e.g., collagen deposition), biomarkers of liver disease (AST, ALT, bilirubin), and markers of inflammation and fibrosis at mRNA and protein levels (e.g., IL6, collagen, TGF-β3, SNAI2).	Preclinical	[160]
Healthy subjects	GB1211	The tablet form of GB1211 had higher bioavailability than the capsule form. Food intake had minimal effects on the pharmacokinetics of the tablet form. 100 mg tablet form was well tolerated and did not cause serious side effects.	Phase 1 clinical trial (GALBA-1; NCT05747573).	[161]
A549 cell line, PBMCs	GB1107, Anti-PD-L1	GB1107 decreased both PD-L1 expression and STAT3 phosphorylation in lung cancer cells in vitro. GB1107 enhanced the cytotoxic activity of T cells against lung cancer cells induced by PD-L1 blockade in vitro.	Preclinical	[162]
Mouse xenograft model of lung cancer	GB1107, Anti-PD-L1	Combination of PD-L1 blockade and GB1107 synergistically reduced tumor growth in vivo, which was accompanied by increased infiltration of CD3^+^ TILs and granzyme B release into tumors.	Preclinical	[162]
Jurkat-Lucia™ TCR-hPD-1, Raji-APC-hPD-L1 cell lines	GB1211, Atezolizumab (anti-PD-L1), Pembrolizumab (anti-PD-1)	GB1211 reduced the binding of Gal-3 to both PD-1 and PD-L1, and restored the binding of atezolizumab and pembrolizumab to PD-L1 and PD-1, respectively.	Preclinical	[163]
LLC1 syngeneic mouse lung cancer model	GB1211, Atezolizumab (anti-PD-L1)	Combination treatment with GB1211 and anti-PD-L1 monoclonal antibody reduced tumor growth in vivo, which was accompanied by an increased percentage of CD8^+^ TILs.	Preclinical	[163]
FSF-TIM3/LysM-Cre+/− mouse model (Myeloid cells with TIM-3 overexpression)	GB1107, Anti-TIM-3	TIM-3 blockade or GB1107 reduced lung inflammation in vivo.	Preclinical	[164]

## Data Availability

Not applicable.
